# TGF-β1 is involved in senescence-related pathways in glomerular endothelial cells via p16 translocation and p21 induction

**DOI:** 10.1038/s41598-021-01150-4

**Published:** 2021-11-04

**Authors:** Sayo Ueda, Tatsuya Tominaga, Arisa Ochi, Akiko Sakurai, Kenji Nishimura, Eriko Shibata, Shu Wakino, Masanori Tamaki, Kojiro Nagai

**Affiliations:** Department of Nephrology, Institute of Biomedical Sciences, Tokushima University Graduate School, 3-18-15, Kuramoto-cho, Tokushima, 770-8503 Japan

**Keywords:** Kidney diseases, Senescence

## Abstract

p16 inhibits cyclin-dependent kinases and regulates senescence-mediated arrest as well as p21. Nuclear p16 promotes G1 cell cycle arrest and cellular senescence. In various glomerular diseases, nuclear p16 expression is associated with disease progression. Therefore, the location of p16 is important. However, the mechanism of p16 trafficking between the nucleus and cytoplasm is yet to be fully investigated. TGF-β1, a major cytokine involved in the development of kidney diseases, can upregulate p21 expression. However, the relationship between TGF-β1 and p16 is poorly understood. Here, we report the role of podocyte TGF-β1 in regulating the p16 behavior in glomerular endothelial cells. We analyzed podocyte-specific TGF-β1 overexpression mice. Although p16 was found in the nuclei of glomerular endothelial cells and led to endothelial cellular senescence, the expression of p16 did not increase in glomeruli. In cultured endothelial cells, TGF-β1 induced nuclear translocation of p16 without increasing its expression. Among human glomerular diseases, p16 was detected in the nuclei of glomerular endothelial cells. In summary, we demonstrated the novel role of podocyte TGF-β1 in managing p16 behavior and cellular senescence in glomeruli, which has clinical relevance for the progression of human glomerular diseases.

## Introduction

p16 is a regulatory motif in cell cycle control, causing specific inhibition of cyclin D/CDK4^1^. By binding to CDK4/6, p16 inhibits cyclin D-CDK4/6 complex formation and CDK4/6-mediated phosphorylation of Rb family members. Expression of p16 maintains the Rb family members in a hypophosphorylated state, which promotes their binding to E2F1 and leads to G1 cell cycle arrest and cellular senescence^2^. Therefore, p16 is involved in pathways regulating senescence-mediated arrest as well as p21^3^. Senescence is a cellular program that induces a stable growth arrest accompanied by distinct phenotypic alterations, including chromatin remodeling and metabolic reprogramming^4,5^. A permanent arrest is effective to ensure that damaged or transformed cells do not perpetuate their genomes. This growth arrest is implemented in the nucleus by the activation of p16/Rb and p53/p21 tumor suppressor networks. In contrast, there is considerable evidence that several neoplasms exhibit significant p16 levels in the cytoplasm^6^. Cytoplasmic p16 has been associated with tumor progression and prognosis in some types of neoplasms. Some roles of cytoplasmic p16 have been suggested, such as in the dissociation of the αvβ3 integrin from focal adhesions^7^. Nevertheless, further work is needed to elucidate the molecular mechanisms involving the cytoplasmic location of p16, its functions, and its connection with oncogene-induced senescence and failure of the p16 tumor suppressor function^8^.

The expression of p16 in kidneys has been evaluated in previous reports. A small number of p16-positive cells, increasing moderately with the age of the donor (up to 0.2% of all cells), was observed in the human kidney. Positive p16 staining was mainly observed in the tubules and was distributed in both the nucleus and cytoplasm. A few p16-positive cells were observed in the Bowman’s capsule of old donors^9,10^. In human chronic kidney diseases, the expression of nuclear p16 is increased in both the tubules and glomeruli in proportion to disease progression^11–13^. Therefore, the location of p16 is important. However, the mechanism of p16 trafficking between the nucleus and cytoplasm has not been thoroughly investigated.

TGF-β1 is well-known as a major cytokine involved in the development of kidney diseases^14,15^. It has been implicated in the regulation of cell proliferation, hypertrophy, apoptosis and fibrogenesis^16^. In particular, TGF-β1 is considered as the master regulator of interstitial fibrosis^17^. On the other hand, the sole effect of TGF-β1 on glomerular pathological changes has not been well studied. Hathaway et al. reported that even 200% normal levels of active TGF-β1 protein in the plasma of non-diabetic mice only caused a slight mesangial expansion without glomerular basement membrane thickening^18^. However, Kopp et al. found that mice transgenic for TGF-β1 under the control of the murine albumin promoter displayed an increased hepatic TGF-β1 expression, eight times increased plasma levels, and florid glomerulosclerosis. These findings indicated that a high level of circulating TGF-β1 could induce an increase in glomerular matrix accumulation^19^. To clarify the role of local TGF-β1 expression, Ghayur et al. induced adenovirus-mediated gene transfer of TGF-β1 mainly in glomerular endothelial cells. Twenty-eight days later, no pathological changes were identified in the glomeruli observed using light microscopy. However, significant proteinuria and foot process effacement was observed. The expressions of podocyte proteins such as nephrin and synaptopodin were decreased^20^. So far, the effect of the long-term locally produced TGF-β1 on glomerular pathological changes has not been clarified.

Many studies have emphasized the importance of TGF-β1 signaling in the regulation of senescence both in vivo and in vitro^21–23^. TGF-β1 can cause oxidative stress-induced activation of the p53/p21pathway and senescence^24^. The Smad pathway located downstream of the TGF-β1 receptors is responsible for upregulating the *p21* gene^25–27^. In contrast, only a few reports suggested that TGF-β1 can increase the expression of p16 even in vitro. Shimoda et al. demonstrated that TGF-β1 induced the expression of p16 in renal fibroblasts^28^. Kandhaya-Pillai et al. reported cell-specific responses of p16 expression to TGF-β1 stimulation. TGF-β1 can upregulate p16 expression by activating the mammalian target of rapamycin pathway in preadipocytes, whereas TGF-β1 can increase p16 expression only under chronic interferon-γ exposure in human fibroblasts^29^. However, to our knowledge, there are no reports on the direct effect of TGF-β1 on the behavior of p16 in vivo.

Therefore, in this study, we investigated the glomerular pathological changes in podocyte-specific TGF-β1 overexpression mice. The aim of this study is to evaluate the effect of long-term locally produced TGF-β1 overexpression in podocytes on the glomerular lesion and senescence-related pathways.

## Results

### Characterization and pathological changes in podocyte-specific TGF-β1 overexpression mice

**Figure 1:**
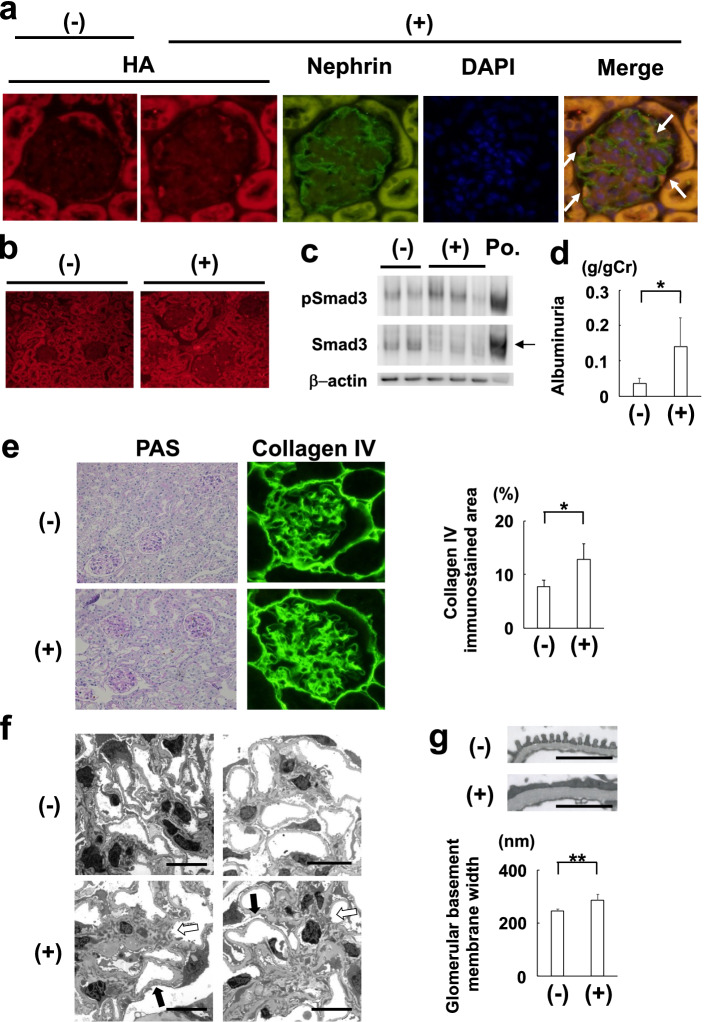
Characterization and pathological changes in podocyte-specific TGF-β1 overexpression mice. (**a**) TGF-β1 was expressed in podocytes. HA-tag was conjugated with bioactive porcine TGF-β1 in PodCre(+) TGF mice. HA-tag merged with nephrin (podocyte marker). (**b**) Diffuse expression of HA-tag conjugated TGF-β1 was observed in the kidney of PodCre(+) TGF mice. (**c**) Representative pictures of western blot analysis of glomeruli protein. The bands immunoblotted with Smad3 increased in size when phosphorylated (arrow). Smad3 was phosphorylated in glomeruli in PodCre(+) TGF mice. (**d**) Urine albumin excretion was significantly increased in PodCre(+) TGF mice. (N = 6 in control mice, N = 8 in PodCre(+) TGF mice). *P < 0.01 (Mann–Whitney’s U test). (**e**) Representative pictures of periodic acid-Schiff (PAS) stain and collagen IV immunohistochemistry in PodCre(+) TGF mice. PodCre(+) TGF mice showed a significant increase in collagen IV immunostained area (N = 6 in control mice, N = 8 in PodCre(+) TGF mice). *P < 0.01 (t-test). (**f**) Representative pictures of electron microscopy. PodCre(+) TGF mice showed mesangial expansion (white arrow) and diffuse foot process effacement (black arrow). Scale bar: 10 µm. (**g**) Representative pictures of glomerular basement membrane by using electron microscopy and quantitative evaluation of glomerular basement membrane width. PodCre(+) TGF mice showed a significant thickening of glomerular basement membrane (N = 3 in control mice, N = 4 in PodCre(+) TGF mice). Scale bar: 2 µm. **P < 0.05 (t-test). (−): control mice. (+): Podocyte-specific TGF-β1 overexpression mice (PodCre(+) TGF mice). Po.: positive control. pSmad3: phosphorylated Smad3. n.s.: not significant.

To evaluate the effect of long-term locally produced TGF-β1 overexpression on the glomerular lesion and senescence-related pathways, we mated *Podocin-Cre* mice with *Cre-dependent TGF-β1* overexpression mice. Double immunostaining analysis of *Podocin-Cre*(+) *TGF-β1* overexpression mice **(**hereafter, PodCre(+) TGF mice**)** using the antibodies against HA tag and nephrin (podocyte marker) revealed that TGF-β1 was expressed in podocytes (Fig. 1a,b). We also confirmed the phosphorylation of Smad3 in the glomeruli of PodCre(+) TGF mice using western blot analysis (Fig. 1c). Albuminuria increased significantly (Fig. 1d), whereas plasma TGF-β1 concentration did not change significantly in PodCre(+) TGF mice at one year of age (mean ± SD, control mice: 63.8 ± 17.7 ng/mL (N = 6), PodCre(+) TGF mice: 77.9 ± 42.3 ng/mL (N = 8)).

Pathologically, in PodCre(+) TGF mice, collagen IV immunostained area revealed that mesangial expansion was significantly induced (Fig. 1e). The mesangial expansion was confirmed by electron microscopy images (Fig. 1f). Diffuse foot process effacement was prominent, and glomerular basement thickening was significant (Fig. 1f,g).

### Induction of cellular senescence in the glomeruli of PodCre(+) TGF mice

**Figure 2:**
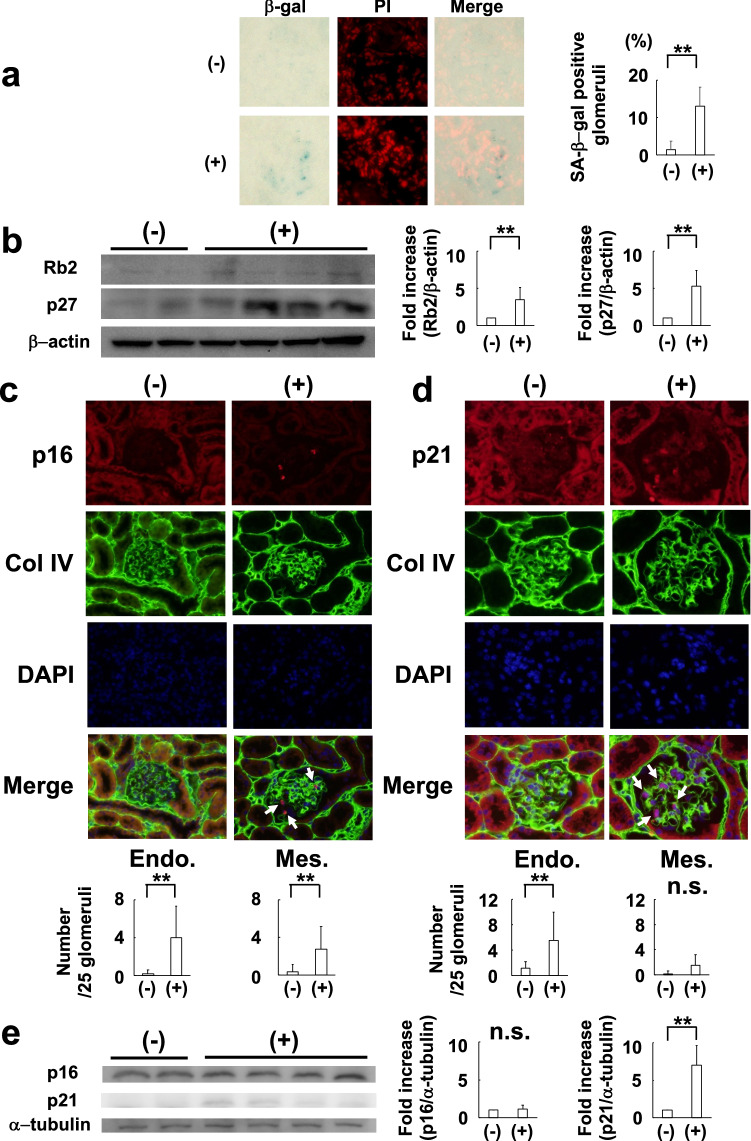
Detection of the markers for cellular senescence in podocyte-specific TGF-β1 overexpression mice. (**a**) Representative pictures of senescence-associated β-galactosidase staining. The nuclei were counterstained with propidium iodide. Senescence-associated β-galactosidase activity was significantly increased in PodCre(+) TGF mice. (N = 3 in control mice, N = 4 in PodCre(+) TGF mice). **P < 0.05 (t-test). (**b**) Representative pictures of western blot analysis of Rb2 and p27 expression in glomeruli. PodCre(+) TGF mice had significant expression levels of Rb2 and p27. (N = 4 in control mice, N = 6 in PodCre(+) TGF mice). **P < 0.05 (t-test). (**c**) Representative pictures of p16 immunohistochemistry. PodCre(+) TGF mice had p16 expression mainly in endothelial cells. PodCre(+) TGF mice showed a significant increase in p16 immunostained nuclei in endothelial and mesangial cells. (N = 6 in control mice, N = 8 in PodCre(+) TGF mice). **P < 0.05 (t-test). (**d**) Representative pictures of p21 immunohistochemistry. PodCre(+) TGF mice had p21 expression mainly in endothelial cells. PodCre(+) TGF mice showed a significant increase in p21 immunostained nuclei in endothelial cells. (N = 6 in control mice, N = 8 in PodCre(+) TGF mice). **P < 0.05 (t-test). (**e**) Representative pictures of western blot analysis of p16 and p21 expression in glomeruli. PodCre(+) TGF mice had a significant expression of p21, but not that of p16. (N = 4 in control mice, N = 6 in PodCre(+) TGF mice). **P < 0.05 (t-test). (−): control mice. (+): Podocyte-specific TGF-β1 overexpression mice (PodCre(+) TGF mice). PI: Propidium iodide. SA-β-gal: Senescence-associated β-galactosidase. Col IV: Collagen IV. Endo.: Endothelial cell. Mes.: Mesangial cell. n.s.: not significant.

To confirm the involvement of TGF-β1 in senescence, we determined the activity of senescence-associated β-galactosidase. β-galactosidase activity was significantly increased in the glomeruli of PodCre(+) TGF mice (Fig. 2a). In addition, we detected the upregulation of senescence-related proteins such as Rb2 and p27 in the glomeruli of PodCre(+) TGF mice (Fig. 2b)^30,31^.

### Expression sites and levels of p16 and p21 in the glomeruli of PodCre(+) TGF mice

We investigated p16 and p21 expression in PodCre(+) TGF mice. The expression site was distinguished by double immunofluorescence staining with the antibody against collagen IV. Both p16 and p21 were significantly immunostained mainly in the nuclei of endothelial cells (Fig. 2c,d). These findings were confirmed by the immunostaining of CD34 (endothelial cell marker) and p16 or p21 using the serial kidney sections (Supplementary Fig. S1 online).

However, western blot analysis showed that compared to control mice, the expression of p16 did not change significantly in the glomeruli of PodCre(+) TGF mice, which seemed inconsistent with the immunohistochemical analysis shown in Fig. 2c. On the other hand, p21 expression was increased significantly in PodCre(+) TGF mice (Fig. 2e).

### The activation of the TGF-β1-Smad3 pathway can induce p21 expression in the late phase, while it can translocate p16 to the nuclei in the early phase in endothelial cells in vitro.

**Figure 3:**
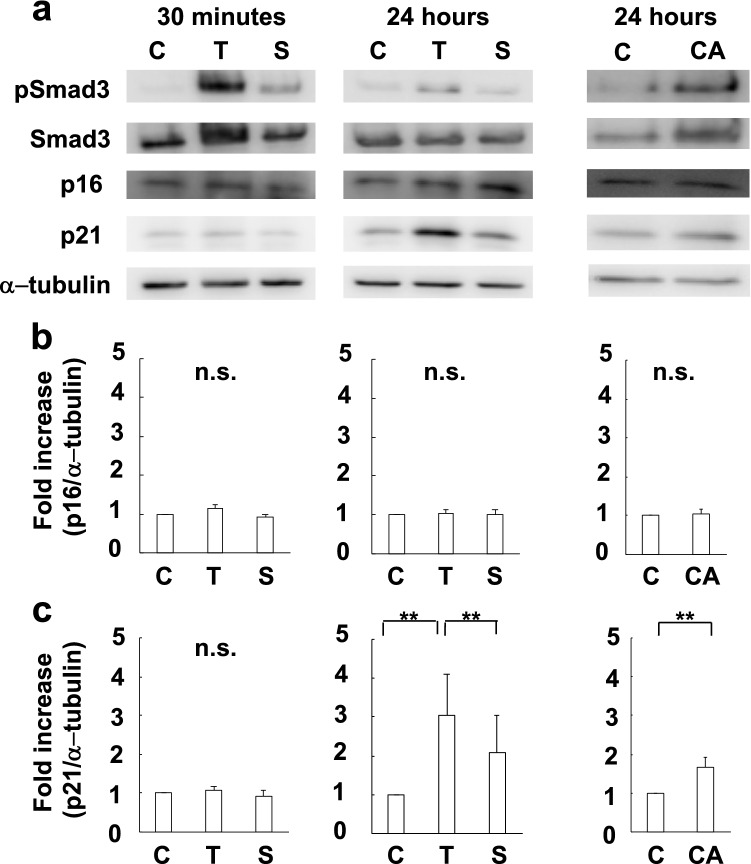
Expression of p16 and p21 induced by the activation of the TGF-β1-Smad3 pathway in endothelial cells. (**a**) Representative pictures of western blot analysis of p16 and p21 expression in endothelial cells induced by the stimulation of the TGF-β1-Smad3 pathway. (**b**, **c**) Activation of the TGF-β1-Smad3 pathway can increase the expression of p21 in 24 h (late phase), but not that of p16 in endothelial cells (N = 3). C: control. T: TGF-β1. S: SB431542. CA: constitutive active Smad3. pSmad3: phosphorylated Smad3. n.s.: not significant. **P < 0.05 (t-test).

Further, we investigated the effect of TGF-β1 on the expression of p16 and p21 in cultured endothelial cells. TGF-β1 could increase p21 expression, not within 30 min, but 24 h (late phase). SB431542, a TGF-β1 receptor antagonist, could suppress the increase in p21 expression. Transfection of constitutive active Smad3 could also increase p21 expression, suggesting p21 expression is controlled by the TGF-β1-Smad3 pathway (Fig. 3a,c). However, TGF-β1 did not affect p16 expression (Fig. 3a,b).

**Figure 4:**
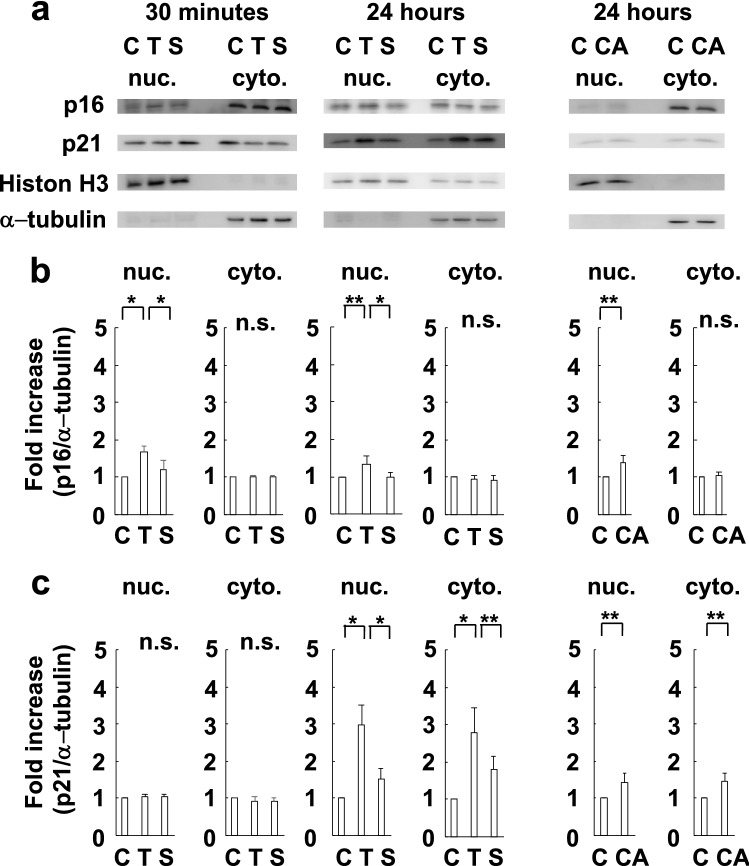
Nuclear translocation of p16 induced by the activation of the TGF-β1-Smad3 pathway in endothelial cells. (**a**) Representative pictures of western blot analysis of p16 and p21 expression in the nucleus and cytoplasm of endothelial cells induced by the stimulation of the TGF-β1-Smad3 pathway. (**b**, **c**) Activation of the TGF-β1-Smad3 pathway can translocate p16 to the nuclei in 30 min (early phase), while it can increase the expression of p21 in endothelial cells in 24 h (late phase) (N = 4). C: control. T: TGF-β1. S: SB431542. CA: constitutive active Smad3. nuc.: nucleus. cyto.: cytoplasm. n.s.: not significant. *P < 0.01. **P < 0.05 (t-test).

Finally, to clarify the inconsistency between in vivo immunohistochemical and western blot analyses, we analyzed the expression of p16 in the nucleus and cytoplasm in cultured endothelial cells separately. p16 was translocated to nuclei by TGF-β1 stimulation in 30 min (early phase), and the effect continued until 24 h later. Analyses using SB431542 and constitutive active Smad3 confirmed that the translocation was induced via the TGF-β1-Smad3 pathway (Fig. 4).

### Expression of β-galactosidase and p16 in the glomeruli of patients with kidney diseases

**Figure 5:**
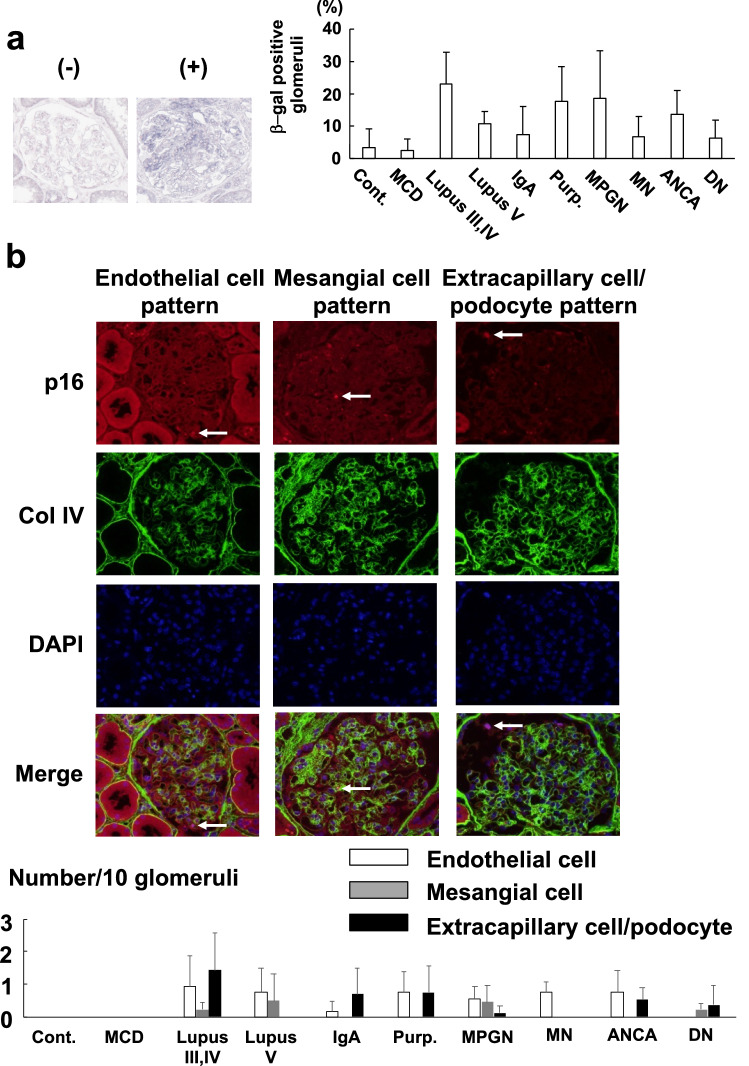
Expression of β-galactosidase and p16 in the glomeruli of patients with kidney diseases. (**a**) β-galactosidase was detected in glomeruli of patients with kidney disease. (**b**) p16 is expressed in endothelial, mesangial cells, and extracapillary cells/podocytes in patients with various human kidney diseases. Representative pictures of each pattern are shown. β-gal: β-galactosidase. Col IV: Collagen IV. Cont.: Control (N = 3). MCD: Minimal change disease (N = 3). Lupus III, IV: Systemic lupus nephritis class III or IV (N = 6). Lupus V: Systemic lupus nephritis class V (N = 3). IgA: IgA nephropathy (N = 6). Purp.: Purpura nephritis (N = 4). MPGN: Membranoproliferative glomerulonephritis (N = 3). MN: Membranous nephropathy (N = 3). ANCA: ANCA glomerulonephritis (N = 6). DN: Diabetic nephropathy (N = 6). White column: Endothelial cells. Grey column: Mesangial cells. Black column: Extracapillary cells and podocytes.

TGF-β1 is involved in the development and progression of various kidney diseases. Therefore, we evaluated the expression of β-galactosidase and p16 using paraffin-embedded human renal biopsy samples. β-galactosidase expression was detected in the glomeruli of patients with kidney diseases, suggesting the induction of cellular senescence (Fig. 5a). In addition, p16 was expressed in endothelial cells of patients with representative glomerulonephritis and nephrotic syndrome except minimal change disease and diabetic nephropathy, suggesting the common pathological significance of p16 in glomerular diseases (Fig. 5b). The expression site was confirmed by double immunofluorescent staining of p16 with CD34 (endothelial cell marker), collagen IV and nephrin (podocyte marker) using human renal biopsy frozen samples (Supplementary Fig. S2 online). Collagen IV was chosen to show the mesangial matrix because there is no specific mesangial cell marker.

## Discussion

In this study, we demonstrated that podocyte TGF-β1 could affect the behavior of p16 in glomerular endothelial cells in vivo and in vitro. We also clarified the pathological and clinical phenotypic changes in kidneys induced by the practical level of TGF-β1 expression.

The most crucial finding in this study is that TGF-β1 was involved in senescence-related pathways via not only p21 but also p16 in glomeruli in vivo and in vitro, because p16 and p21 are major molecules responsible for cellular senescence. The relationship between the TGF-β1-Smad3 pathway and p21 has been well clarified^24–27^. However, the direct effect of the TGF-β1-Smad3 pathway on p16 behavior had not been investigated, especially in vivo. Several reports suggested the contribution of TGF-β1 to cellular senescence via a p16 mediated mechanism in vitro^32^. However, to our knowledge, the connection between TGF-β1 and p16 in cellular senescence had not been proved in vivo. We reveal the novel mechanism of TGF-β1 involvement in p16 behavior, which is different from the interaction between TGF-β1 and p21. TGF-β1 could not increase the expression of p16 in glomeruli, but could induce nuclear translocation of p16 in glomerular endothelial cells.

p16 expression in kidney tubules and interstitial cells has been studied in mouse models and human kidney diseases with respect to the progression of kidney fibrosis and aging of kidney^33^. For example, high phosphate activates senescence in renal tubular cells through distinct but interconnected mechanisms: upregulation of p16/p21, elevation of plasminogen activator inhibitor-1 and downregulation of Klotho, followed by fibrosis^34^. In addition, the acute kidney injury-to-chronic kidney disease transition may involve a wide range of mechanisms, including the action of scar-forming myofibroblasts, microvascular rarefaction, mitochondrial dysfunction, or cell cycle arrest by the involvement of the epigenetic, gene, and protein alterations leading to common final signaling pathways such as TGF-β1, p16, Wnt/β-catenin pathway involved in renal aging^35^. However, to our knowledge, p16 expression in each cell comprised of glomeruli has not been thoroughly investigated. In human kidney diseases such as IgA nephropathy, nephrotic syndrome, and diabetic kidney disease, as well as aging kidney, p16 expression was found in mesangial, endothelial cells, and podocytes^10–13^. On the other hand, there were few reports evaluating p16 expression in glomeruli using the in vivo animal kidney disease model. Aratani et al. showed that p16 is involved in radiation-induced kidney disease by immunohistochemical analysis^36^. In the diabetic kidney disease model, western blot analysis revealed that p16 expression increased significantly in glomeruli^37–39^. So far, our animal study is the first to evaluate p16 expression in glomeruli quantitatively using both western blot and immunohistochemical analyses in vivo. We revealed that the increase in nuclei positive immunostaining of p16 does not always coincide with the upregulation of p16 expression in vivo.

In this investigation, we would like to clarify the sole and glomerulus-specific TGF-β1 effect on glomerular diseases. The role of TGF-β1, especially in the interstitial area of the kidney, has been well evaluated using conventional fibrosis models such as the UUO model^40^. This model causes a relatively rapid progression of fibrosis. However, the model does not cause glomerulopathy, and we cannot determine the role of TGF-β1 in glomerular diseases using this model. Diabetic nephropathy is one of the representative and important glomerular diseases. The animal models of diabetic nephropathy display glomerular lesions such as mesangial matrix expansion, glomerular basement membrane thickening, and mild tubulointerstitial damage^41^. However, these changes are induced by hyperglycemia. Hyperglycemia triggers many types of cytokines, chemokines, and several signaling pathways including protein kinase C cascade, Janus kinase/signal transducer and activator of transcription signaling, mitogen-activated protein kinase, mammalian target of rapamycin, and Smad^42^. Although TGF-β1 is involved in the progression of diabetic nephropathy^43,44^, it is just one of the cytokines involved in the progression of diabetic nephropathy. We cannot determine the sole effect of TGF-β1 on glomerular diseases. Therefore, our model has the strength to clarify the sole effect of TGF-β1 on glomerular diseases in vivo.

Several studies have previously reported the role of TGF-β1 by using the TGF-β1 overexpression mouse model. Kopp et al. reported that TGF-β1 overexpression in the liver can cause kidney glomerulosclerosis^19^. However, this glomerulosclerosis model has an eight times higher expression. Hathaway et al. revealed that TGF-β1 expression level could influence the kidney manifestation in the mice, especially under diabetic conditions^18^. These findings mean that TGF-β1 can cause glomerulosclerosis if its concentration is exceptionally high or any other risk factors such as cytokines and metabolic conditions concur with TGF-β1 stimulation. In human kidney diseases, the reports evaluating plasma TGF-β1 concentration were limited. Plasma TGF-β1 concentration can increase according to kidney dysfunction or diabetic kidney injury. In older community-dwelling adults, the levels of median plasma TGF-β1 were higher for those with eGFR < 60 mL/min/1.73 m^2^ compared to those with eGFR > 60 mL/min/1.73 m^2^ ^45^. In patients with diabetic kidney disease, baseline median plasma TGF-β1 level was two times higher in participants with progressive kidney disease than participants whose kidney disease had not progressed^46^. Iwano et al. investigated intraglomerular TGF-β1 mRNA in patients with human kidney diseases. TGF-β1 mRNA was significantly elevated in patients with mesangial proliferative glomerulonephritis having a moderate increase in the mesangial matrix, diabetic nephropathy and lupus nephritis compared to participants with normal glomeruli. TGF-β1 mRNA expression levels in patients with diffuse proliferative lupus nephritis were more than five times higher than those with normal glomeruli^47^. Unfortunately, in our mouse model, we could not estimate the local expression level of total TGF-β1 in glomeruli quantitatively, because we used an overexpression model of porcine TGF-β1, which does not have the same potency as mouse TGF-β1. However, our mice had similar plasma TGF-β1 concentration as the control mice, which is consistent with the previous report using the same mice^48^. Therefore, we believe that our mouse model clarifies the development of primary background lesions in various human kidney diseases, because TGF-β1 is involved in the development and progression of these diseases^14,15^. Our mice would represent the early stage of glomerular lesions considering pathological changes such as mild mesangial expansion, podocyte injury, and albuminuria. Moreover, we could show that the practical level of TGF-β1 per se causes the expression of senescence-related molecules in the nuclei of glomerular endothelial cells. Therefore, endothelial senescence can be triggered in the early stage of various human kidney diseases, as p16 expression was found in the nuclei of endothelial cells in human renal biopsy samples from many kinds of kidney diseases in this study (Fig. 5b, Supplementary Fig. S2 online), consistent with the previous findings of glomerular TGF-β1 mRNA expression in patients with kidney diaseases^47^. Endothelial senescence could be one of the important mechanisms in the progression of arteriosclerosis in glomeruli^24^. Probably, in addition to the TGF-β1-related basic alterations of pathology and molecular behavior in mesangial, endothelial cells, and podocytes shown in this study, various cytokines and growth factors modify kidney lesions, followed by the establishment of complex and disease-specific kidney manifestation.

In this study, we investigated the phenotype in podocyte-specific TGF-β1 overexpression mice, which have glomerulus-specific TGF-β1 overexpression. Regarding the expression site of TGF-β1 in the glomeruli of human kidney diseases, both Yamamoto et al. and Ito et al. reported that TGF-β1 is expressed in podocytes as well as mesangial, endothelial cells of glomeruli in patients with proliferative nephritis^14,49^. In patients with advanced diabetic nephropathy, TGF-β1 is immunostained in both matrix and remnant cells of glomeruli^43,50^. The limitation of our mouse model is that the podocyte-specific TGF-β1 overexpression mouse model can only partly explain the pathogenic role of TGF-β1 in these glomerulopathies. However, considering the phenotype of the mice having TGF-β1 overexpression in glomerular endothelial cells for 28 days in the previous report^20^, which resembled our results in terms of podocyte injury and proteinuria, TGF-β1 could cause a podocyte-endothelial crosstalk^51^. In addition, it is technically impossible to investigate the effect of glomerular endothelial-specific or mesangial-specific TGF-β1 overexpression in vivo.

In conclusion, we found the involvement of the TGF-β1-Smad3 pathway in the behavior of p16 in glomeruli in vivo and in vitro. These findings will be one of the common and novel molecular mechanisms in the progression of various human kidney diseases.

## Methods

### Ethics statement

All clinical investigations were conducted according to the principles expressed in the Declaration of Helsinki. All patients provided informed written consent for participation in and publication of the study. The animal experiment was carried out in compliance with the ARRIVE guidelines. All experiments were performed following the institutional guidelines and regulations of Tokushima University. The study, including this human study and the animal experiments, was approved by the Research Ethics Committee of Tokushima University.

### Subjects

Renal biopsy samples derived from different human glomerular diseases such as minimal change disease, lupus nephritis, IgA nephropathy, purpura nephritis, membranous proliferative glomerulonephritis, membranous nephropathy, ANCA glomerulonephritis, and diabetic nephropathy diagnosed at Tokushima University Hospital were analyzed in this study. Renal biopsy tissues were fixed in Dubosque-Brazil’s solution. Biopsy samples from patients with asymptomatic hematuria served as controls and showed minor glomerular abnormalities and negative immunofluorescence. The profiles of control and patients with human kidney diseases are shown in Table 1.

**Table 1:** The characteristics of the subjects included in this study.

	Number (N)	Females (N)	Age (mean ± SD) (years)
Control	3	2	36.0 ± 24.3
MCD	3	2	52.7 ± 23.2
Lupus class III or IV	6	4	38.5 ± 13.5
Lupus class V	3	3	35.7 ± 7.6
IgA nephropathy	6	4	43.3 ± 8.9
Purpura nephritis	4	1	62.0 ± 12.8
MPGN	3	2	65.3 ± 16.1
MN	3	1	52.3 ± 13.2
ANCA glomerulonephritis	6	4	78.2 ± 7.2
Diabetic nephropathy	6	1	62.8 ± 11.8

MCD: Minimal change disease. Lupus: Systemic lupus nephritis. MPGN: Membranoproliferative glomerulonephritis. MN: Membranous nephropathy.

### Mice

*Podocin-Cre* mice and *Cre-dependent HA-tagged TGF-*β*1* overexpression mice were obtained from The Jackson Laboratory (Bar Harbor, ME, USA). Before starting this experiment, all mice were backcrossed 10 times to ICR (CLEA Japan Inc., Tokyo, Japan). In PodCre(+) TGF mice, HA-tag was conjugated with bioactive porcine TGF-β1. Urine and plasma were collected from the mice, and the mice were sacrificed at one year of age to analyze the pathological changes in the kidney.

### Immunohistochemical analysis

Immunohistochemical analysis was performed on paraffin-embedded sections using the indirect immunohistochemistry procedure with rabbit anti-p16 (sc-1207, Santa Cruz Biotechnology, Dallas, TX, USA), anti-p21 (ab109199, Abcam, Cambridge, UK), anti-HA (3724, Cell Signaling Technology, Beverly, MA, USA), anti-CD34 (ab81289, Abcam), goat anti-collagen IV (1340–01, SouthernBiotech, Birmingham, AL, USA), chicken anti-β-galactosidase (AB-3403, Merck Millipore, Billerica, MA, USA) and sheep anti-nephrin (AF4269, R&D Systems, Minneapolis, MN, USA) antibodies. For p16, p21, and collagen IV immunostaining, sections were pretreated with proteinase K (19131, QIAGEN K.K., Tokyo, Japan). For immunostaining with the other antigens, sections were pretreated with citrate buffer (pH 6.0). In the double immunofluorescent analysis of frozen human renal biopsy samples with cell-specific markers, a mouse monoclonal antibody against p16 (F-12) (sc-1661, Santa Cruz Biotechnology) was employed. Following the first antibody, the sections were incubated with Alexa Fluor 488 or 594-conjugated donkey anti-rabbit antibody (A21206, A21207, Invitrogen, Grand Island, NY, USA) against p16, p21, HA, and CD34; Alexa Fluor 488-conjugated donkey anti-goat antibody (A11055, Invitrogen) against collagen IV; Alexa Fluor 488-conjugated donkey anti-sheep antibody (A11015, Invitrogen) against nephrin; Alexa Fluor 594-conjugated donkey anti-mouse antibody (A32744, Invitrogen) against p16 (F-12). Nuclei were visualized by DAPI (D523, DOJINDO Laboratories, Kumamoto, Japan). For β-galactosidase immunostaining of paraffin-embedded human tissue sections and CD34 immunostaining of paraffin-embedded mouse tissue sections, Avidin/Biotin Blocking System (SIG-31126, BioLegend Inc., San Diego, CA, USA), biotin-conjugated anti-chicken antibody (BA-9010, Vector Laboratories Inc., Burlingame, CA, USA), biotin-conjugated anti-rabbit antibody, HRP-conjugated streptavidin (426011, 426061, Nichirei Biosciences Inc., Tokyo, Japan), alkaline phosphatase-conjugated streptavidin (S921, Thermo Fisher Scientific Inc., Waltham, MA, USA), BCIP/NBT, and DAB substrate kit (SK-5400, SK-4100, Vector Laboratories Inc.) were used. The immunohistochemical signal was quantified using Image J^52^. The values were expressed as a percentage of glomerular surface area occupied by the collagen IV immunostained area. The positively stained nuclei number was counted for p16 and p21 immunostaining. Mean values were calculated using data obtained from six to eight mice. For each sample, 25 glomerular profiles were measured. In human renal biopsy samples, 10 glomerular profiles per subject were analyzed.

### Senescence-associated β-galactosidase activity

Senescence-associated β-galactosidase was detected using Senescence β-Galactosidase Staining Kit (9860, Cell Signaling Technology) according to the manufacturer’s instruction. Nuclei were visualized by propidium iodide (P3566, Thermo Fisher Scientific Inc.). For each sample, 25 glomerular profiles were measured. Mean values were calculated using data obtained from three to four mice.

### Western blotting

In vivo, glomeruli from one-year-old mice were collected by magnetic beads-based isolation^53^. Briefly, transcardiac perfusion was performed using phosphate-buffered saline containing precleaned beads (Dynabeads, Invitrogen). The perfused renal cortex was briefly digested with collagenase A (Roche, Basel, Switzerland) and deoxyribonuclease I (Invitrogen), and the glomeruli stuffed with beads were isolated by DYNAL (Invitrogen). Glomeruli were lysed using Mammalian Cell Extraction Kit (BioVision Inc., Milpitas, CA, USA). Lysates of glomeruli were subjected to SDS-PAGE and immunoblotted with the following primary antibodies: rabbit antibody against p16 (ab108439, Abcam), p21 (ab109199, Abcam), phospho-Smad3 (ab52903, Abcam), and Smad3 (ab28379, Abcam), and mouse antibody against Rb2 (610262, BD Biosciences, San Jose, CA, USA), p27 (610241, BD Biosciences), α-tubulin, and β-actin (T6199, A5316, Sigma-Aldrich, St. Louis, MO, USA). In vitro, lysates of cultured endothelial cells were immunoblotted with the antibodies mentioned above and goat anti-Histon H3 (sc-8654, Santa Cruz Biotechnology). Immobilon ECL Ultra Western HRP Substrate (Merck Millipore) was used to detect the blotting signals using LAS-3000 (FUJIFILM, Tokyo, Japan). The immunohistochemical signal was quantified using Image J^52^. Mean values were calculated using data obtained from four to six mice or three to four independent in vitro experiments.

### Electron microscopy

Tissues used for electron microscopy were fixed with 2.5% glutaraldehyde. We entrusted electron microscopy analysis to a specialized company (BML Inc. Tokyo, Japan.)^54^. Glomerular basement membrane width was measured using Image J^52^. Mean values were calculated using data obtained from three to four mice. For each sample, six glomerular basement membrane widths were measured.

### Urine albumin and creatinine

Urinary albumin and creatinine were determined using Albuwell M and Creatinine Companion kits (Exocell Inc., Philadelphia, PA, USA).

### Plasma TGF-β1 concentration

Mouse plasma was obtained using heparinized hematocrit tubes (Drummmond scientific company, Broomall, PA, USA). Plasma TGF-β1 concentration was analyzed by quantikine ELISA (R and D systems).

### Endothelial cell culture

Mouse immortalized endothelial cell line, TKD2 (RIKEN BioResource Research Center, Ibaraki, Japan) was maintained in growth medium (Dulbecco's modified Eagle's medium (DMEM); Sigma-Aldrich) supplemented with 1 mM glutamine, penicillin at 100 units/mL, streptomycin at 100 µg/mL (Invitrogen) and 10% fetal bovine serum (Sigma-Aldrich) at 33 degrees centigrade. The cells (1.2 × 10^6^/well) were plated in 9 cm culture dishes (Fine Plus International Ltd., Kyoto, Japan). Twenty-four hours later, the cells were serum-starved in DMEM containing 0.5% bovine serum albumin (Sigma-Aldrich) and pretreated with DMSO (Sigma-Aldrich) or SB431542 (1 µM; Cayman Chemical, Michigan, USA), a potent and specific inhibitor of TGF-β type I receptor, for two hours. They were stimulated with TGF-β1 (10 ng/ mL; PeproTech, Rocky Hill, NJ, USA). Cell lysates were harvested using Mammalian Cell Extraction Kit or Nuclear/Cytosol Fractionation Kit (BioVision Inc., Milpitas, CA, USA), 30 min or 24 h after stimulation. In a transfection experiment, control or constitutively active Smad3 expression vector was kindly provided by Dr. J. Oh (Korea University)^55^. Plasmids were transfected using Novagen® GeneJuice® Transfection Reagents (Merck Millipore) according to the manufacturer’s protocol. Cell lysates were harvested 24 h after transfection.

### Statistical analysis

All values are expressed as mean ± SD. Statistical analysis was performed using SPSS for Windows version 13.0 (SPSS Inc., Chicago, IL, USA). If data were normally distributed, the results were compared using Student’s t-test or Welch’s t-test. Non-normal data were analyzed by Mann–Whitney’s U test. F-test was used for comparing the factors of total deviation, and the significance was set at *P* less than 0.05.

## Supplementary Information

Supplementary Information.
