# Supplementary material for: TGF-β1 is involved in senescence-related pathways in glomerular endothelial cells via p16 translocation and p21 induction
Source: Sci Rep. 2021 Nov 4;11:21643. doi: 10.1038/s41598-021-01150-4 (PMC8569175; doi:10.1038/s41598-021-01150-4)
Supplement: Supplementary file 1 — Supplementary Information. [file 41598_2021_1150_MOESM1_ESM.pdf]

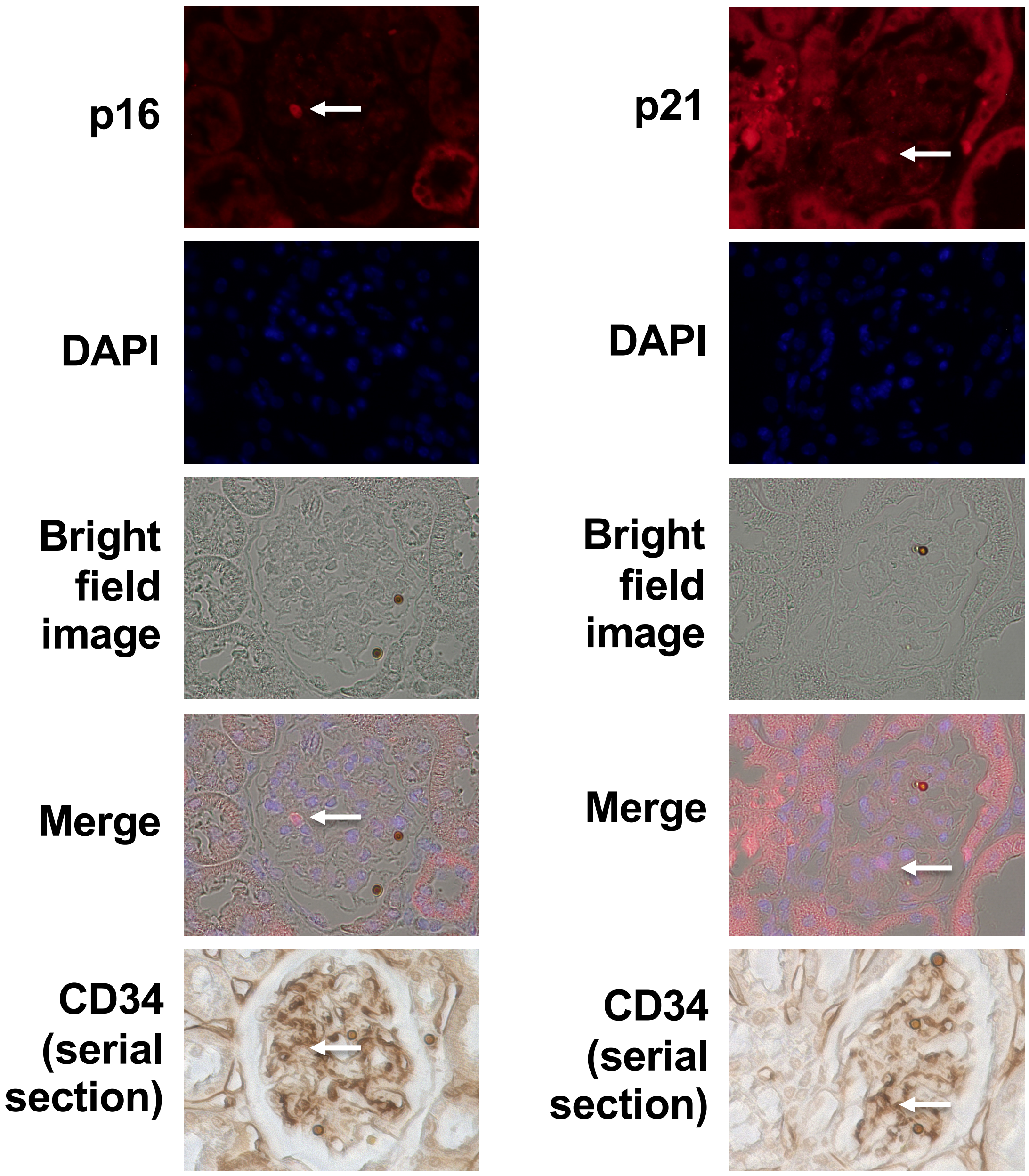


**Supplementary Fig. S1.**

p16 or p21 immunostained nuclei were detected mainly in endothelial cells in podocyte-specific TGF-β1 overexpression mice.

Representative pictures of p16 or p21 and CD34 (endothelial cell marker) immunostaining using the serial kidney sections.

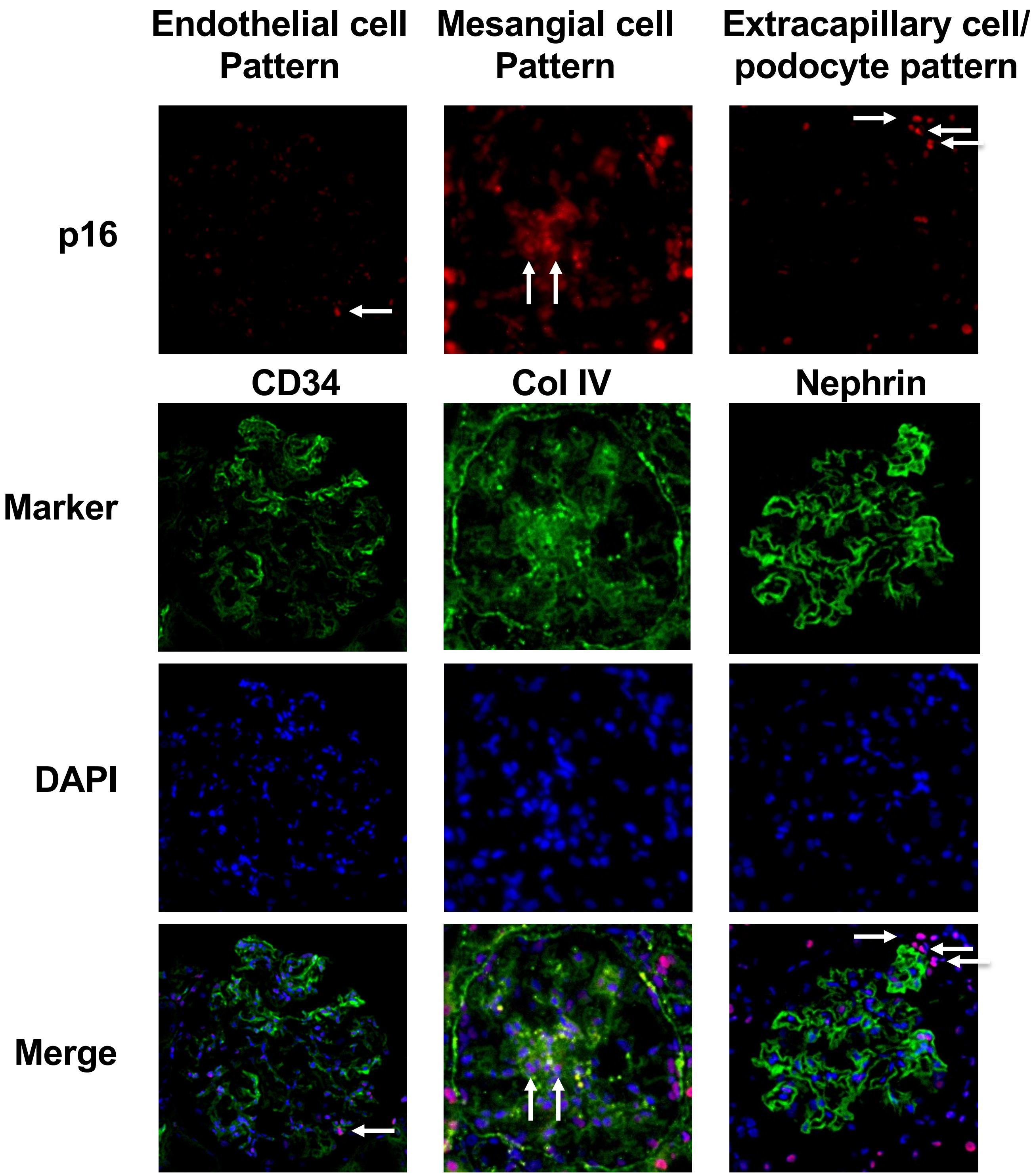


**Supplementary Fig. S2.**

p16 immunostained nuclei were detected in endothelial, mesangial cells and extracapillary cells/podocytes in the renal biopsy samples of patients with various human kidney diseases.

Representative pictures of p16 and CD34, collagen IV, nephrin (endothelial cell marker, mesangial matrix marker, podocyte marker) immunostaining using frozen human renal biopsy samples.

**Supplementary Fig. S3.**

Unprocessed versions of the figure images.

Figure 1c

Two sets of proteins were applied by a mirror image style.

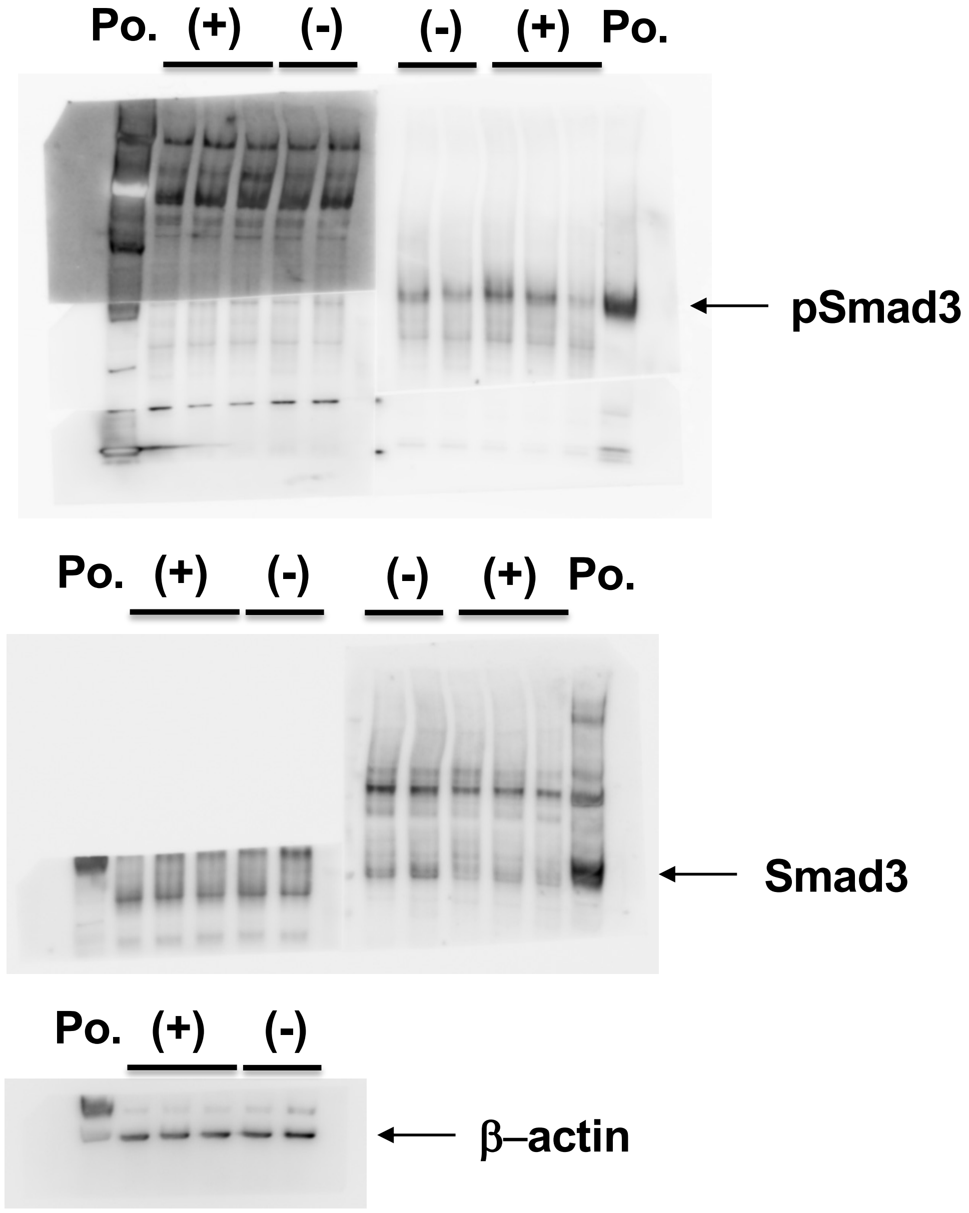

Figure 2b

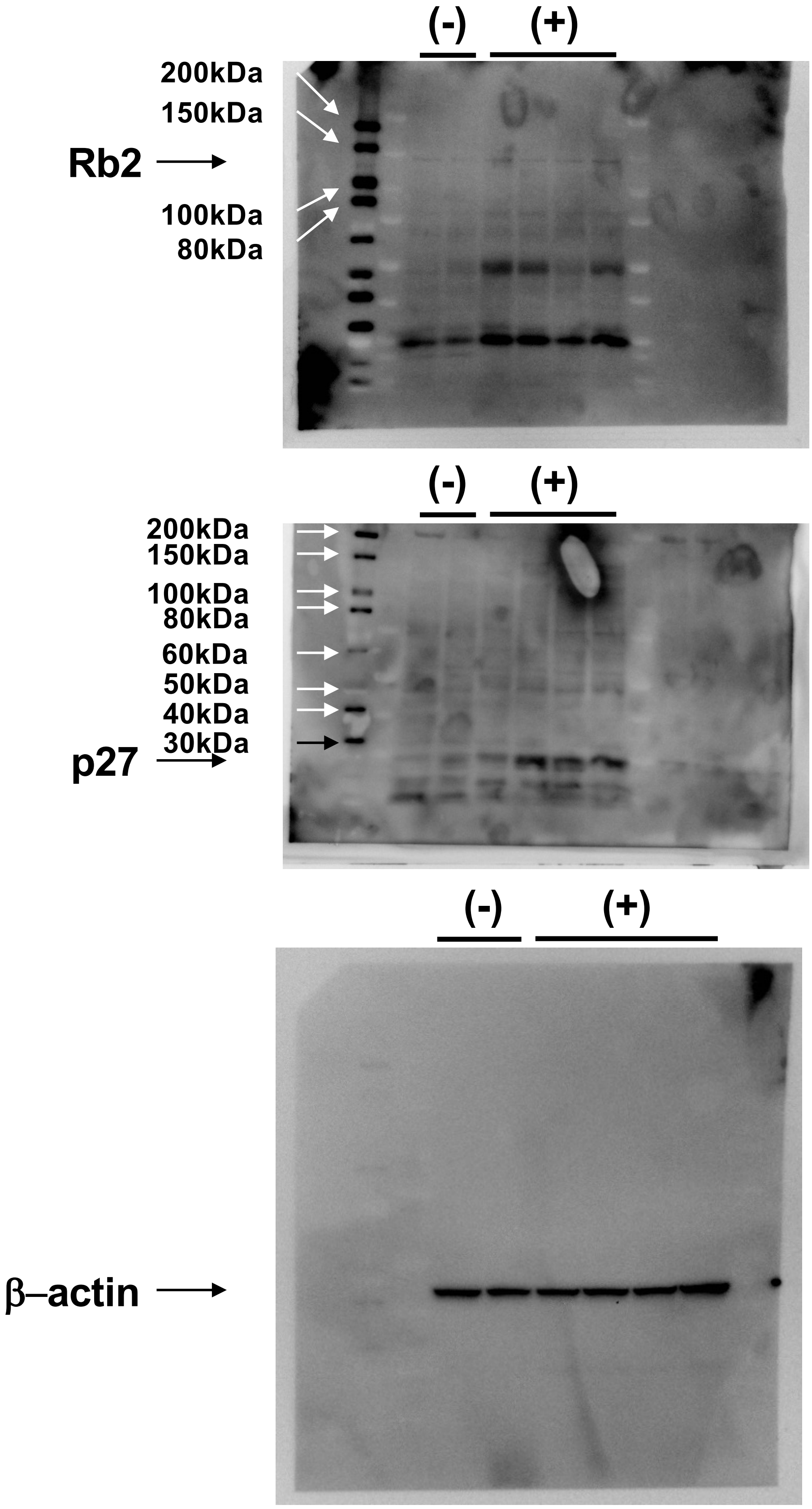
(-)
(+)
200kDa
150kDa
Rb2
100kDa
80kDa
(-)
(+)
200kDa
150kDa
100kDa
80kDa
60kDa
50kDa
40kDa
30kDa
p27
(-)
(+)
β–actin

Figure 2e

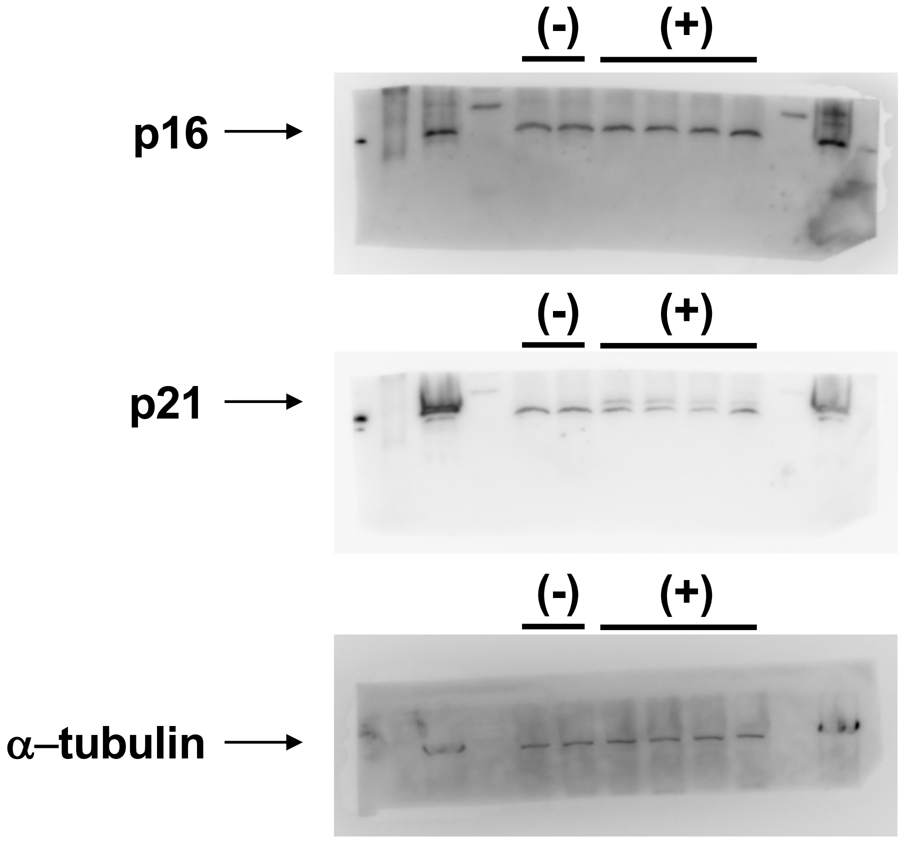

Figure 3a

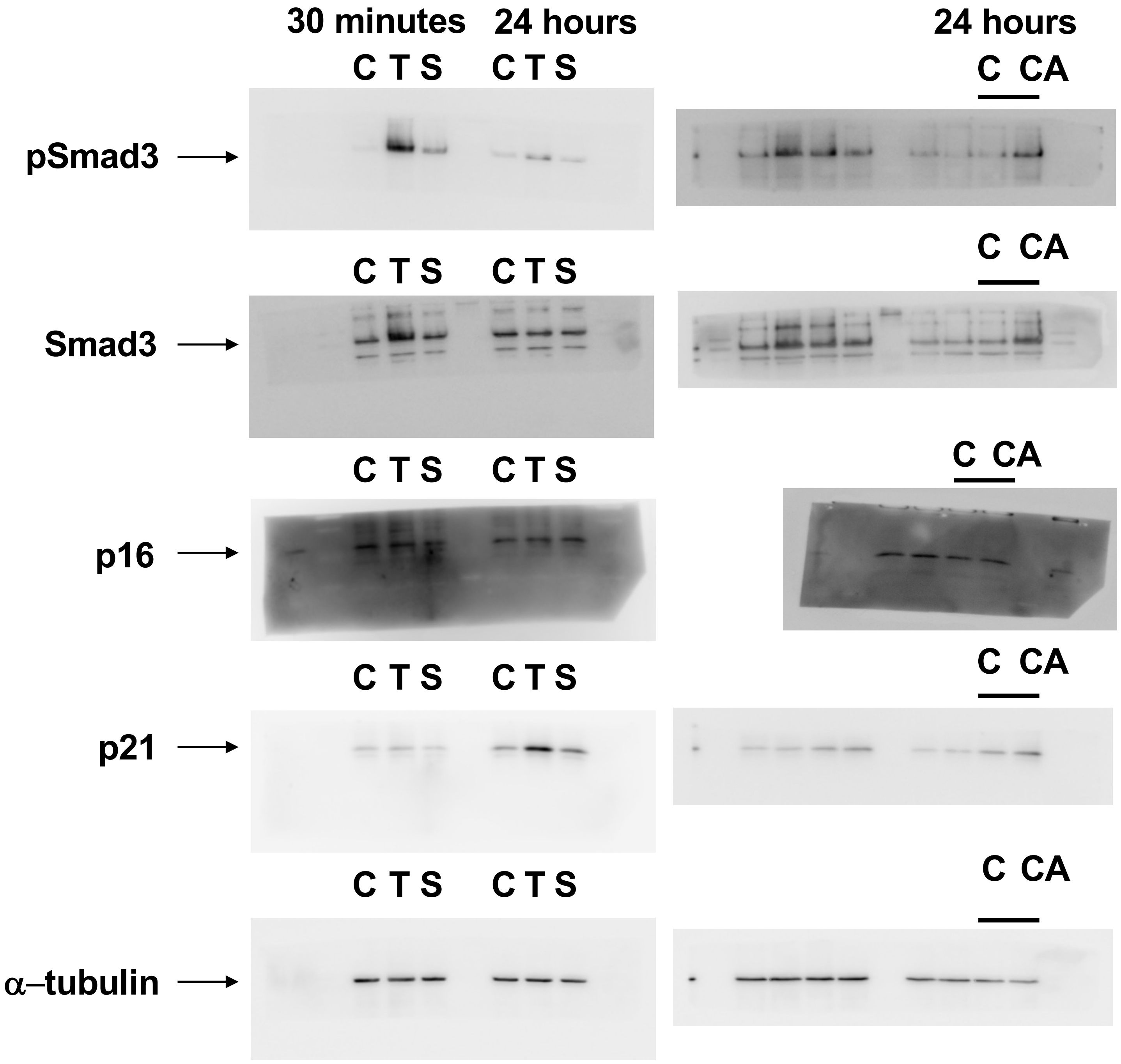

30 minutes 24 hours
C T S C T S
24 hours
C CA
pSmad3
Smad3
p16
p21
α–tubulin

Figure 4a

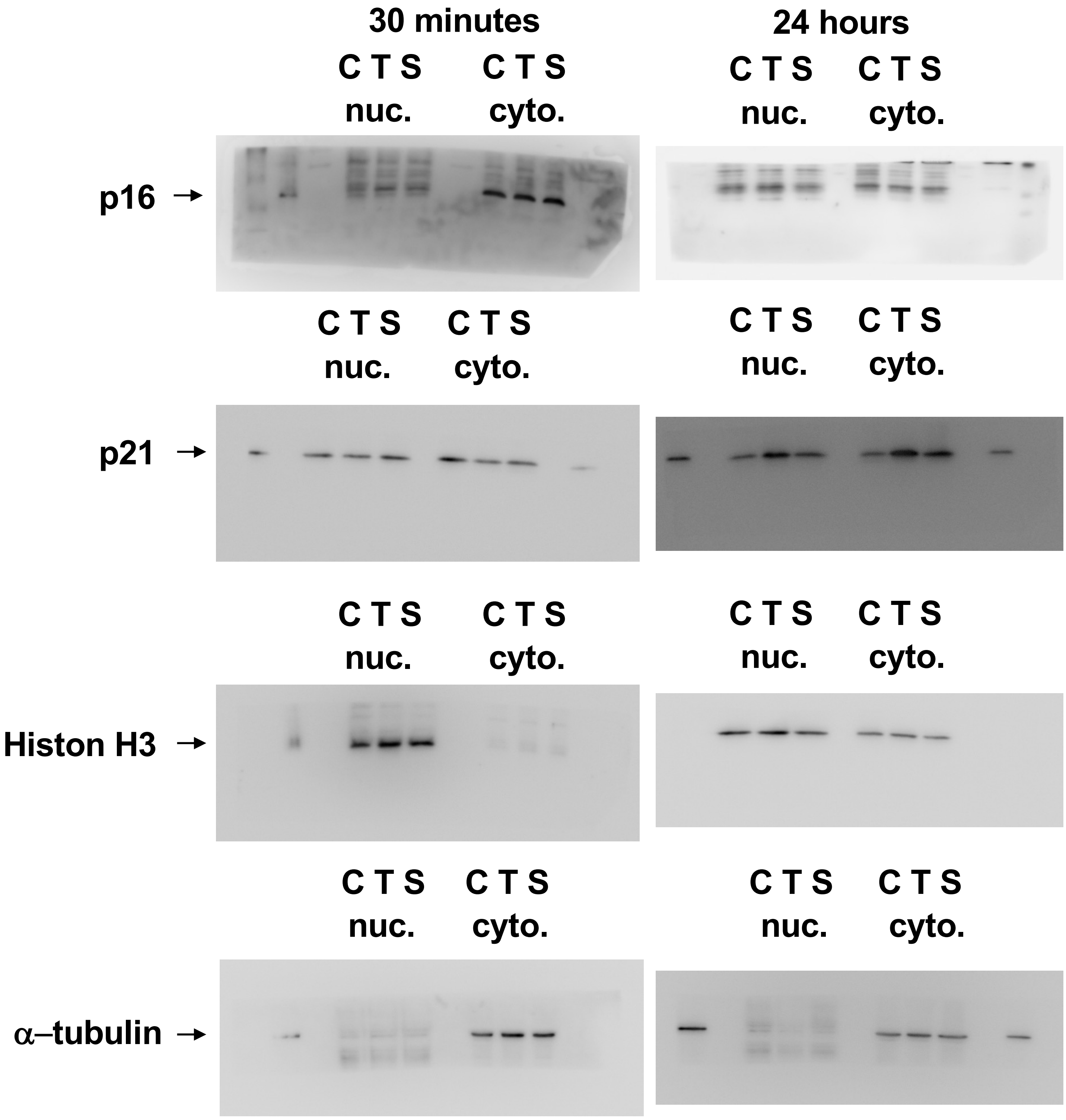
30 minutes
24 hours
C T S
C T S
nuc.
cyto.
p16
p21
Histon H3
α–tubulin

Figure 4a

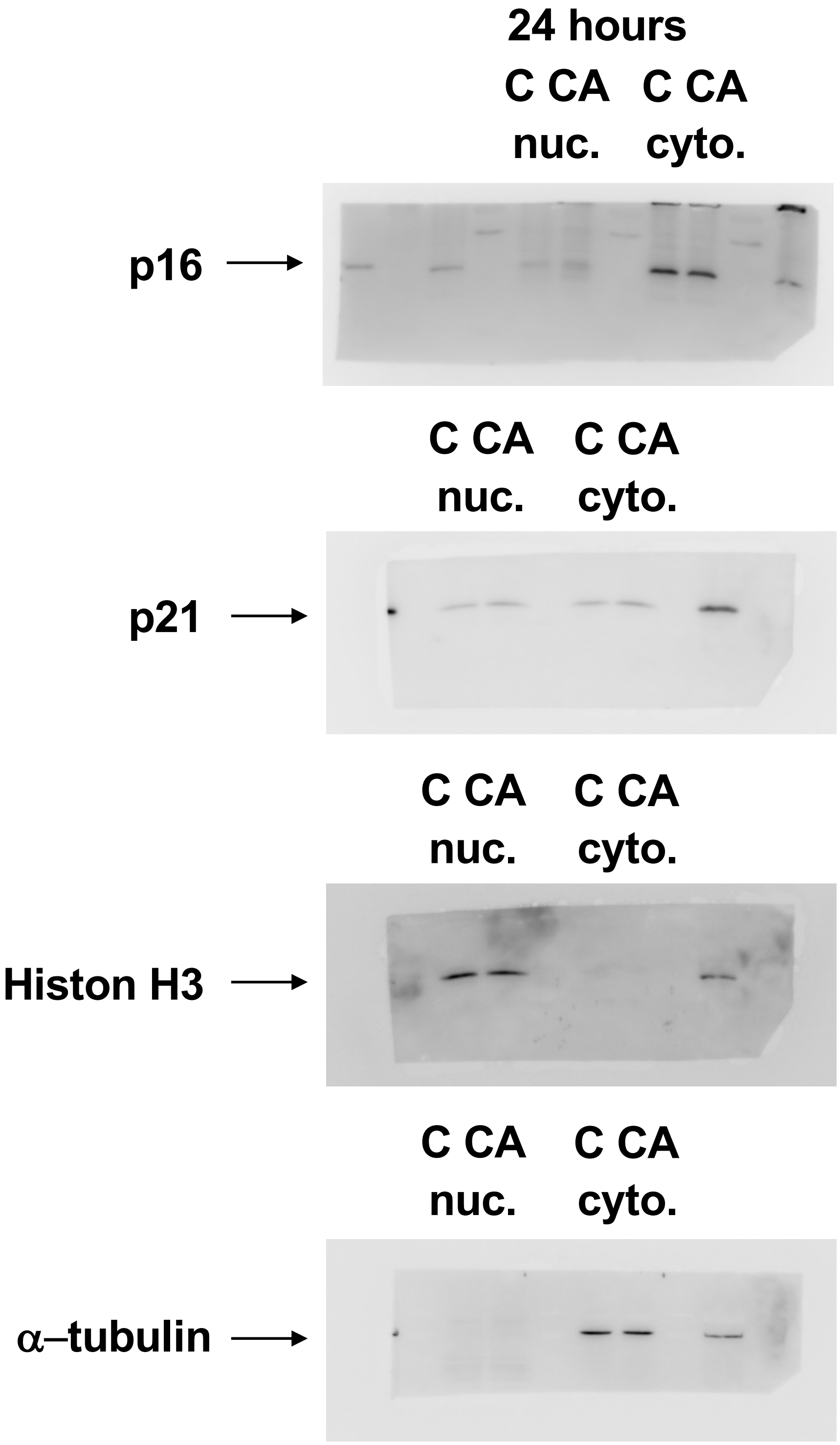
24 hours
C CA C CA
nuc. cyto.
p16
C CA C CA
nuc. cyto.
p21
C CA C CA
nuc. cyto.
Histon H3
C CA C CA
nuc. cyto.
α–tubulin
